# False negative rate at ^18^F-FDG PET/CT in para-aortic lymphnode involvement in patients with locally advanced cervical cancer: impact of PET technology

**DOI:** 10.1186/s12885-021-07821-9

**Published:** 2021-02-06

**Authors:** Sebastien Gouy, Veronika Seebacher, Cyrus Chargari, Marie Terroir, Serena Grimaldi, Anna Ilenko, Amandine Maulard, Catherine Genestie, Alexandra Leary, Patricia Pautier, Philippe Morice, Désirée Deandreis

**Affiliations:** 1 Department of Gynecologic Surgery, Gustave Roussy and Paris Saclay, 114 Rue Edouard Vaillant, Villejuif, France; 2 University Paris Sud, Orsay, France; 3 Unit INSERM 1030, 114 Rue Edouard Vaillant, Villejuif, France; 4 Department of Radiotherapy and Brachytherapy Unit, Gustave Roussy and Paris Saclay, 114 Rue Edouard Vaillant, Villejuif, France; 5 Effets biologiques des rayonnements, Institut de Recherche Biomédicale des Armées, Bretigny-sur-Orge, France; 6 Department of Nuclear Medicine and Endocrine Oncology, Gustave Roussy and Paris Saclay, 114 Rue Edouard Vaillant, 94800 Villejuif, France; 7 Department of Pathology, Gustave Roussy and Paris Saclay, 114 Rue Edouard Vaillant, Villejuif, France; 8 Department of Medical Oncology, Gustave Roussy and Paris Saclay, 114 Rue Edouard Vaillant, Villejuif, France; 9 Department of Medical Sciences, Nuclear Medicine Division, the University of Turin, C.so Dogliotti, 14 10126 Turin, Italy

**Keywords:** LACC, Cervical cancer, PET/CT, TOF, Para-aortic lymph node, FDG

## Abstract

**Background:**

The identification of factors responsible for false negative (FN) rate at ^18^F- Fluorodeoxyglucose (FDG) Positron Emission Tomography /Computed Tomography (PET/CT) in para-aortic (PA) lymph nodes in the presurgical staging of patients with locally advanced cervical cancer (LACC) is challenging. The aim of this study was to evaluate the impact of PET/CT technology.

**Methods:**

A total of 240 consecutive patients with LACC (International Federation of Gynecology and Obstetrics, FIGO, stage IB2-IVA) and negative Magnetic Resonance Imaging (MRI) and/or Computed Tomography (CT) and negative ^18^F-FDG PET/CT in the PA region, undergoing laparoscopic PA lymphadenectomy before chemoradiotherapy were included. The FN rate in patients studied with Time of flight (TOF) PET/CT (TOF PET) or non-Time of flight PET/CT (no-TOF PET) technology was retrospectively compared.

**Results:**

Patients presented with FIGO stage IB (*n* = 78), stage IIA-B (*n* = 134), stage III (*n* = 18) and stage IVa (*n* = 10), squamous cell carcinoma (*n* = 191) and adenocarcinoma (*n* = 49). 141/240 patients were evaluated with no-TOF PET/CT and 99/240 with TOF PET/CT. Twenty-two patients (9%) had PA nodal involvement at histological analysis and considered PET/CT FN findings. The FN rate was 8.5% for no-TOF PET and 10% for TOF PET subgroup respectively (*p* = 0.98). Ninety patients (38%) presented with pelvic node uptakes at PET/CT. The FN rate in the PA region was 18% (16/90) and 4% (6/150) in patients with and without pelvic node involvement at PET/CT respectively (19 vs 3% for no-TOF PET and 17 vs 5% for TOF PET subgroup).

**Conclusions:**

In LACC, FN rate in PA lymph nodes detection is a clinical issue even for modern PET/CT, especially in patients with pelvic uptake. Surgical lymphadenectomy should be performed in case of negative PET/CT at PA level in these patients, while it could be discussed in the absence of pelvic uptake.

## Background

The therapeutic approach in locally advanced cervical cancer (LACC) is a challenge and based on tumor volume, nodal metastasis, and clinical stage [1]. In LACC (i.e. FIGO stage IB2 to IVA) chemoradiation in association with pelvic External Beam Radiation Therapy (EBRT) is recommended in case of metastatic pelvic but negative Para-Aortic (PA) lymph nodes; in case of metastatic PA lymph nodes, an extended radiation field to PA region is applied [1, 2].

Therefore, imaging techniques with high diagnostic accuracy are fundamental for disease staging. Diagnostic imaging in cervical cancer includes abdomino-pelvic Magnetic Resonance Imaging (MRI), Computed Tomography (CT) scan and ^18^F-Fluorodeoxyglucose (FDG) Positron Emission Tomography /Computed Tomography (PET/CT) ^18^F-FDG PET/CT [3–6].

^18^F-FDG PET/CT may help to rule out loco-regional lymph nodes and distant metastasis through a whole-body examination. Indeed, recent guidelines recommend PET/CT over CT in initial staging of LACC to detect extra pelvic disease and for EBRT plan delineation [1]. However, in patients with LACC, high false negative rate for PET/CT in detecting metastatic PA lymph nodes has been reported in several published studies [7–11]. For this reason, surgical staging is still considered in patients with negative presurgical ^18^F-FDG PET/CT at PA level [12]. During the last years, we assisted to a rapid development of PET technology. In particular, the introduction of Time of Flight (TOF) technique using crystal materials with relatively high time resolution should theoretically increase the sensitivity and consequently lesion detectability [13–15].

The aim of this study is to determine the impact of TOF PET/CT vs no-TOF PET/CT technology on false negative rate in PA lymph node detection in the presurgical staging of patients with LACC.

## Methods

### Study design and patient enrolment

All consecutive patients with locally advanced cervical cancer (LACC) according to FIGO classification (stage IB2-IVA) treated between 2007 and 2015 at Gustave Roussy (GR), with negative morphological imaging (abdomino-pelvic MRI in most cases or CT scan and pelvic MRI) and negative ^18^F-FDG PET/CT in the para-aortic area (PA), undergoing laparoscopic PA lymphadenectomy before chemoradiotherapy were included. Patients with a poor prognosis according to histological subtype or peritoneal carcinomatosis were excluded. Institutional review board of Gustave Roussy approved the study and waived the need to obtain informed consent.

The surgery included the removal of PA nodes from the aortic bifurcation to the left renal vein. PA above and below the inferior mesenteric artery, preaortic, superficial intercavoaortic and precaval groups were removed. Pelvic nodes were not resected because included in the radiotherapy field.

All patients were treated with pelvic external beam radiation therapy (45 to 50 Gy) associated to a concomitant cisplatin-based chemotherapy protocol after surgery. Patients with histologically proven PA node metastasis after staging laparoscopic surgery were treated with pelvic and extended to PA region EBRT (45 to 60 Gy) with concomitant cisplatin chemotherapy.

**Fig. 1:**
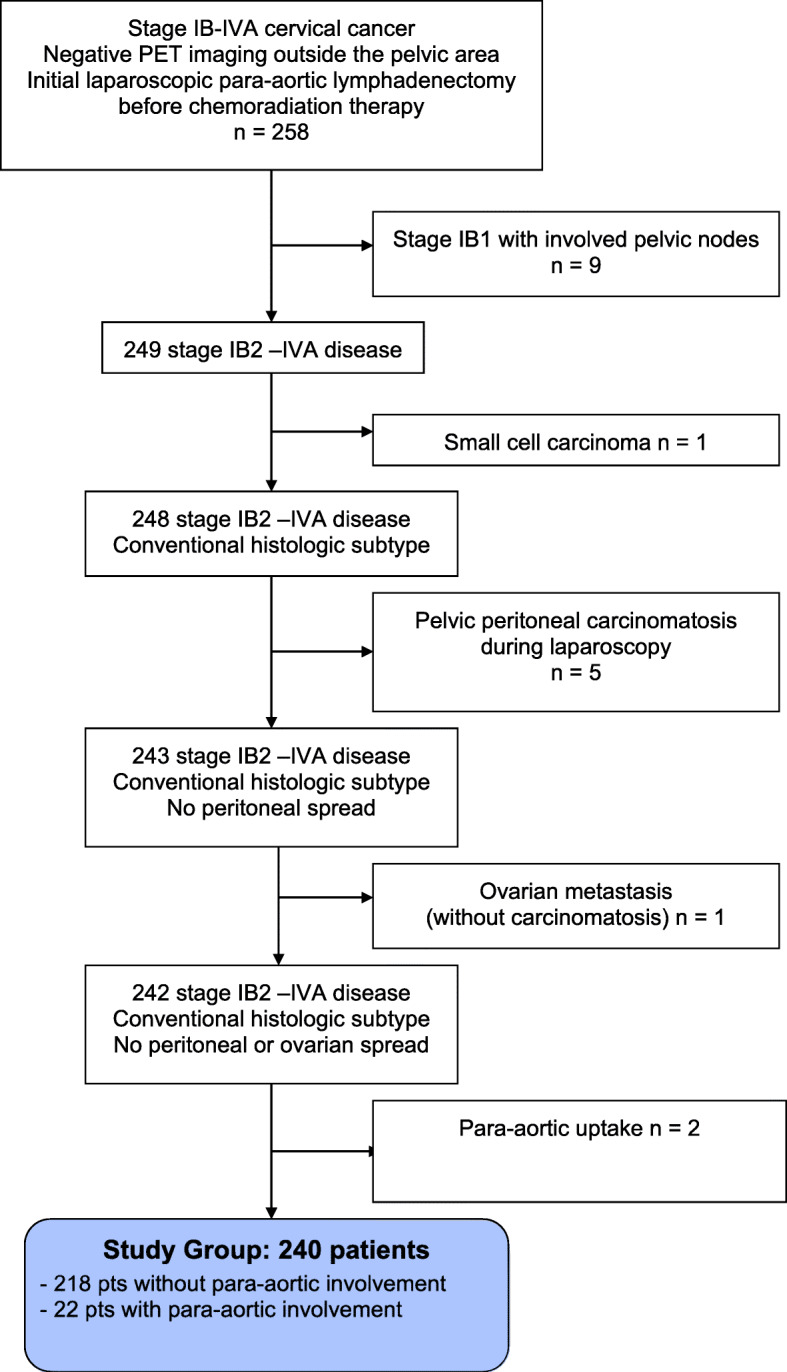
Consolidated standards of reporting Trials (CONSORT) DIAGRAM of the study

A total of 258 patients were initially evaluated. Eighteen patients were excluded from analysis because of a final IB1 staging with involved pelvic nodes (*n* = 9), small cell carcinoma histology (*n* = 1), discovery of peritoneal carcinomatosis during laparoscopy (*n* = 5), ovarian metastasis (*n* = 1) and dubious findings for lymph node metastases in the para-aortic region at PET/CT (*n* = 2) **(**Fig. 1**)**. In the end, a total of 240 patients were considered for the analysis and two subgroups of patients were retrospectively identified according to the PET/CT technology used: patients studied with TOF PET/CT and patients studied with no-TOF PET/CT.

### ^18^F-FDG pet/CT

All the patients underwent a ^18^F-FDG PET/CT before surgery. One hundred and eighteen patients underwent ^18^F-FDG PET/CT at Gustave Roussy and the remaining 122 in others different Nuclear Medicine Services in France according to guidelines [16]. After 6 h of fasting, patients performed images acquisition 60 min after the administration of a median activity of 254 MBq of ^18^F-FDG (range: 137–591 MBq). Median glycaemia before PET/CT was 4.6 mmol/L (range:0.74–10.4).

On the basis of constructor characteristics and reconstruction algorithms, PET/CT scanners were divided into two categories: TOF PET/CT (TOF PET) using 3D coincidence algorithms for image reconstruction and including TOF system (PET/CT Gemini-TF Philips, PET/CT Biograph mCT20-mCT40 Siemens, PET/CT Discovery 690 General Electric Healthcare, PET/CT Discovery 710 General Electric Healthcare) and no-TOF PET/CT (no-TOF PET) with 2D-3D coincidence algorithms for image reconstruction and not provided with TOF system (PET/CT Biograph 6-Siemens, PET/CT Gemini GXL).

### Statistical analyses

Patient characteristics were reported as relative/absolute frequencies for categorical covariates and as median (range) for continuous ones. Their potential differences, when stratified as TOF-PET vs. no-TOF PET, were inferred by the Fisher’s exact test for categorical variables and the Mann-Whitney test for continuous ones. All reported *p*-values were obtained by the two-sided exact method, at the conventional 5% significance level. Data were analysed by R 3.6.3 (R Foundation for Statistical Computing, Vienna-A, http://www.R-project.org).

## Results

### Patient characteristics

The median age was 45 years (21–68). Among 240 patients, 78/240 presented with stage IB, 134/240 with stage IIA-B, 18/240 with stage III and 10/240 with stage IVa of the disease respectively. One hundred ninety-one patients (*n* = 191) had squamous cell carcinoma and 49 had adenocarcinoma. Patient Body Mass Index (BMI) ranged from 14 to 43 Kg/m2 with a median of 23 Kg/m2.

**Fig. 2:**
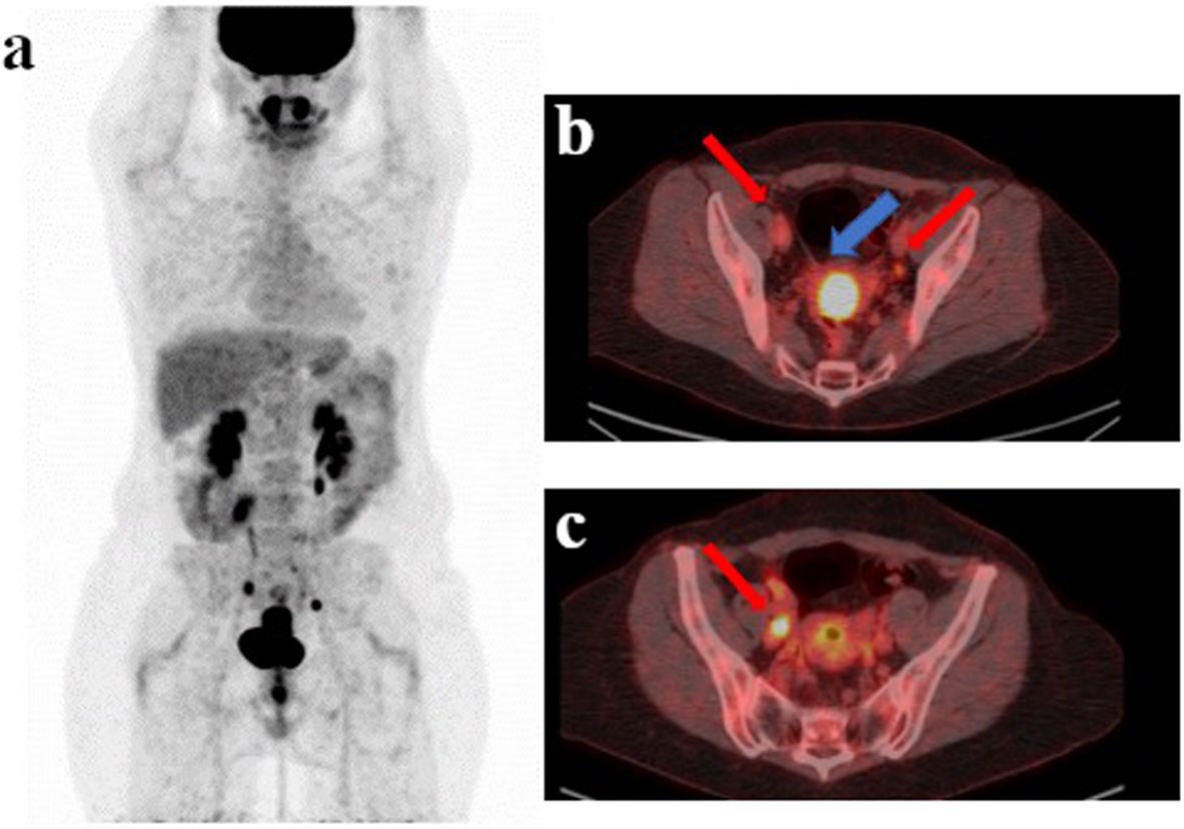
This is a case (**a**) of a patient with stage IIB cervical cancer (**b** blu arrow) studied with TOF PET/CT presenting pelvic pathological lymph nodes (red arrow) located in right (**b**, **c** red arrow: 2 lymph nodes), in the left pelvic region (**c** red arrow: 1 lymph node) and in the common iliac region. No significant uptake was present in the para-aortic region at. A single macrometastase in the para-aortic region was detected at final histology

Before surgery, ^18^F-FDG PET showed uptake in pelvic lymph nodes in 90/240 (38%) patients. The uptake was unilateral in 57/90 (63%) patients, located in the right (45%) and in the left region (51%) respectively (3.5% missing data). All patients with unilateral uptake presented with ≤2 positive lymph nodes at ^18^FDG PET/CT. Pelvic uptake was bilateral in 33/90 (37%) of patients **(**Fig. 2**).** Thirty patients presented with ≤2 positive lymph nodes at ^18^FDG PET/CT by side, while the remaining 3 three patients presented with > 2 positive lymph nodes (5 + 2 in 1 case, 2 + 3 in 1 case and 1 + 3 in 1 case in the left and right region respectively). In the remaining 150/240 (62%) there was no uptake in the pelvic region. In regard to surgical laparoscopy techniques, 235 patients underwent a retroperitoneal approach and five a transperitoneal approach. One patient had conversion to laparotomy. The mean delay between ^18^F-FDG PET/CT and surgery was 15.5 days (range: 1–56). The median number of removed PA lymph nodes per patient was 15 (range: 2–40). Twenty-two patients (9%) had para-aortic nodal involvement at histological analysis considered as FN PET findings with a median of 2 metastatic lymph nodes per-patient (range: 1–8). Twenty-one patients had squamous carcinoma and 1/22 had a clear cell adenocarcinoma subtype. In 13/22 (59%) the size of the biggest PA involved node was > 5 mm while in the remaining 9/22 (41%) the biggest size was ≤5 mm. Among the 22 patients with PA metastases, 16 presented pelvic ^18^F-FDG uptake at PET/CT while the remaining 6 patients did not. The FN rate in the PA region was 18% (16/90) and 4% (6/150) in patients with and without pelvic lymph node uptake respectively. The FN rate was 16% (9/57) and 21% (7/33) in patients with unilateral and bilateral pelvic uptake respectively. Patient population characteristics are summarized in Table 1.

**Table 1:** Patient Population characteristics

Characteristics	No of patients (*N* = 240)	%
Age in years, median (range)	45 (21–68)	
Tumor stage (FIGO classification)		
IB2	78	33
IIa	13	5
IIb	121	50
III	18	7
IVa	10	4
BMI in kg/m^2^, median (range)	23 (14–43)	
Histologic subtype		
Adenocarcinoma	49	20
Squamous cell carcinoma	191	80
Pelvic node uptake during PET imaging		
No	150	62
Yes	90	38
Unilateral pelvic uptake	57	63
Bilateral pelvic uptake	33	37
Delay in days between PET-surgery, median (range)	15.5 (1–56)	
No of para-aortic nodes removed, median (range)	15 (2–40)	
No of patients with positive para-aortic nodes	22	9
Size of the biggest para-aortic nodes involved, mm		
< 5	9	41
> 5	13	59

*Abbreviations*: *FIGO* International Federation of Gynecology and Obstetrics, *BMI* Body Mass Index, *PET* Positron Emission Tomography

### TOF vs no-TOF PET/CT analysis

In relation to PET tomograph technology, 141/240 patients were evaluated with no-TOF PET/CT (73 at GR and 68 in other centers) and 99/240 with TOF PET/CT (45 at GR and the remaining 54 in other centers). Patient population characteristics of the two groups are summarized in Table 2. Patients studied with TOF PET were slightly older than no-TOF PET group (median age 48 vs 45 years; *p = 0.009*). The majority of patients were classified as stage IIb, 73/141 (51.8%) compared to 48/99 (48.5%) in no-TOF and TOF PET group respectively *(p = 0.36)*. Before surgery, ^18^F-FDG PET showed uptake in pelvic lymph nodes in 48/141 (34%) and 42/99 (42%) patients evaluated with no-TOF and TOF PET/CT scan respectively *(p = 0.50)*. The median number of PA lymph nodes removed was 15 (range: 2–39) and 16 (range: 3–40) in the group of patients evaluated with no-TOF and TOF PET/CT *(p = 0.25)* respectively. Amongst the first group, 12/141 (8.5%) presented with metastatic PA lymph nodes at histology compared to 10/99 (10%) in the second group *(p = 0.98).* The maximum size of metastatic PA lymph nodes was > 5 mm in 7/12 (58.3%) and in 6/10 (60%) patients and ≤ 5 mm in 5/12 (41.7%) and 4/10 (40%) respectively in patients evaluated with no-TOF and TOF PET/CT *(p = 0.48)*. The FN rate was 19% (9/48) and 17% (7/42) in patients with pelvic uptake and 3% (3/93) and 5% (3/57) in patients without pelvic uptake in the no-TOF and TOF PET group. FN rate was 16% (5/32) and 16% (4/25) in cases of unilateral pelvic uptake vs 25% (4/16) and 18% (3/17) in cases of bilateral uptake in the no-TOF and TOF PET group (Table 3).

**Table 2:** Patient Population characteristics according to the two groups (no-TOF PET and TOF PET technology)

Characteristics	no-TOF (*N* = 141)	TOF (*N* = 99)	*p*-value
Age in years, median (range)	45 (21–68)	48 (22–65)	0.009
Tumor stage (FIGO classification)			
IB2	50 (35.5%)	28 (28.3%)	
IIa	5 (3.5%)	8 (8.1%)	
IIb	73 (51.8%)	48 (48.5%)	0.36
III	8 (5.7%)	10 (10.1%)	
IVa	5 (3.5%)	5 (5%)	
BMI in kg/m2, median (range)	23 (14–42)	23 (16–35)	0.68
Histologic subtype			
Adenocarcinoma	31 (22%)	18 (19%)	0.62
Squamous cell carcinoma	110 (78%)	81 (81%)	
Median size primary tumor	48 mm (20–100)	45.5 mm (10–85)	0.32
Median SUVmax primary tumor	9.8 (3–43)	14.6 (5.6–35)	< 0.001
Pelvic node uptake during PET imaging			
No	93 (66%)	57 (58%)	
Yes	48 (34%)	42 (42%)	0.5
Unilateral pelvic uptake	32	25	
Bilateral pelvic uptake	16	17	0.83
Delay in days between PET-surgery, median (range)	15 (1–56)	17 (2–56)	0.22
No of para-aortic nodes removed, median (range)	15 (2–39)	16 (3–40)	0.25
No of patients with positive para-aortic nodes	12 (8.5%)	10 (10%)	0.98
Size of the biggest para-aortic nodes involved, mm			
< 5	5	4	
> 5	7	6	0.48

*Abbreviations: TOF* Time of Flight, *FIGO* International Federation of Gynecology and Obstetrics, *BMI* Body Mass Index, *SUV* Standardized Uptake Value, *PET* Positron Emission Tomography

**Table 3:** False negative rate of ^18^F-FDG PET/CT in para-aortic region according to pelvic uptake

Pelvic node uptake	All patients (***N*** = 22)	no-TOF (***N*** = 12)	TOF (***N*** = 10)
**NO**	4% (6/150)	3% (3/93)	5% (3/57)
**YES**	18% (16/90)	19% (9/48)	17% (7/42)
**-unilateral**	16% (9/57)	16% (5/32)	16% (4/25)
**-bilateral**	21% (7/33)	25% (4/16)	18% (3/17)

*Abbreviations: TOF* Time of Flight, ^*18*^*F-FDG* ^18^F- Fluorodeoxyglucose (FDG), *PET/CT* Positron Emission Tomography /Computed Tomography

## Discussion

PA lymph node metastases occur in 10–25% of patients with LACC and are correlated with pelvic, common iliac nodes involvement and larger primary tumor size [17, 18]. The detection of metastatic PA lymph nodes is a fundamental step to define the correct therapeutic approach in LACC and to improve patient outcome [19–22]. False negative rates for ^18^F-FDG PET and PET/CT in PA areas have been reported in several studies ranging from 5 to 17% [7, 11].

The identification of factors responsible for false negative PET/CT findings in PA region in LACC is challenging. In the first studies on this topic, false negative rate was mainly related to lymph node size < 5 mm considered beyond the machine resolution. In the study by Roh et al. including patients with stages IA to IVA PET sensitivity was 38% increasing to 52% in case of lymph nodes > 5 mm and 65% in the case of lymph nodes > 10 mm [7].

The use of hybrid machines such as PET/CT has greatly improved the accuracy of the technique allowing a better localisation and characterisation of abdominal FDG uptakes [23]. However, results among the studies are variable and even in more recent studies false negative rate of ^18^F-FDG PET/CT remains high up to 22%, in particular in early-stage disease [24–27]. The retrospective study by Leblanc et al. including 125 patients with cervical cancer in stage IB2 –IIA with negative pre-operative CT scans or MRI, FDG PET or PET/CT showed a sensitivity of 33% in PA lymph nodes detection with false negative rate of 67% [10]. A recent metanalysis including 29 studies focused on ^18^F-FDG PET alone and PET/CT reported a pooled sensitivity of 0.72 in detecting both pelvic and PA lymph nodes, 0.83 in case of locally advanced disease and 0.41 in early-stage disease [6]. Another recent recent metanalysis from Yu et al. including 14 studies evaluated specifically the diagnostic performance of PET/CT for the detection of PA lymph nodes [28]. The authors reported a pooled sensitivity and a pooled specificity of 0.71 (95% CI: 0.54–0.83) and 0.97 (95% CI: 0.93–0.98) respectively and a pooled positive likelihood ratio (PLR) and negative likelihood ratio NLR (NLR) of 21.53 and 0.30, respectively. The authors concluded that ^18^F-FDG PET is an important imaging method for the detection of PA lymph nodes but underline the limitations of the included studies, mostly retrospective and with low sample size. The ACRIN6671 trial reported a sensitivity for ^18^F-FDG PET with contrast enhanced CT of 0.83 in the detection of pelvic lymph nodes decreasing to 0.50 for PA lymph nodes [29].

In a previous multicentric prospective study evaluating 237 patients in the same setting, our group demonstrated ^18^F-FDG PET/CT false negative rate of 12% and the results supported the conclusion that PA lymph node dissection should be performed in LACC in case of negative ^18^F-FDG PET/CT in PA region for a proper patient staging [11].

The impact of PET device has never been taken into account in order to evaluate and to compare accuracy in this scenario. The aim of this study was to evaluate the impact of PET/CT technology on false negative rate in PA lymph node detection in LACC staging. In the last decades there have been multiple advances in PET technology with improvement in cancer imaging [30]. The introduction of Time of Flight (TOF) technique using crystal materials with relatively high time resolution should increase the sensitivity and (consequently) lesion detectability especially in overweight patients [31, 32]. This technology is useful in clinical practice, allowing shorter examinations, reduction of the radiation dose to the patient, successful scanning of overweight patients, clearer characterisation of low uptake areas and visualization of smaller lesions [31, 32].

Our data show that the false negative rate in PA lymph node detection in patients evaluated by PET/CT scan without TOF system integration compared to PET/CT with TOF integration is comparable (8.5 vs 10%; *p* = 0.98). These findings suggest that even if the sensitivity or lesion detectability is supposed to be higher in modern PET/CT scanner the limitation of the technique to assess PA lymph node is still a clinical issue in this specific clinical setting. The resolution of the PET/CT technology with TOF is considered between 4 and 6 mm [13, 31, 32]. Considering lesion size, in our study 59% of the metastatic lymph nodes detected at histology presented with a diameter > 5 mm with a similar distribution in the two groups. In our opinion, the size cannot be considered the only cause of the false negative rates of ^18^F-FDG PET/CT. Some studies suggest that ^18^F-FDG uptake can be influenced by tumoral histotype in primary and metastatic lesions, with higher ^18^F-FDG uptake in squamous cell compared to non-squamous cell tumors. In the study of Lin et al. nodal metastasis was less likely to be detected by ^18^F-FDG-PET in patients with early-stage adenocarcinoma compared to squamous carcinoma [33]. Nevertheless, in our study almost 80% of the patients and 21/22 patients with false negative ^18^F FDG PET/CT presented with squamous cell carcinoma. The para-aortic lymph nodes are close to the urinary tract and ^18^F-FDG physiological urinary elimination can hide small ^18^F-FDG avid lesions. In this case, the use of hybrid imaging such as PET/MRI and contrast enhanced diagnostic CT coupled with PET might better differentiate between pathological and physiological uptake, but well-designed trials are warranted to confirm this assumption [34]. Patient BMI should be also taken into account because in overweight patients the quality of images can be affected and the detection rate reduced, but the BMI in the two groups of our study was similar.

Finally, patients with pelvic pathological nodes metastases, compared to patients without pathological pelvic nodes, have a higher risk to develop PA metastases [17, 18]. In our study the percentage of pelvic positive lymph nodes in TOF PET compared to no-TOF PET group (42% vs 34%) was not statistically different (*p* = 0.5). FN rate in PA region was higher in case of pelvic uptake at ^18^F-FDG PET/CT, especially when bilateral, compared to negative pelvic uptake cases. These data are confirmed in both no-TOF and TOF PET groups with FN rate of 19% vs 17 and 3% vs 5% respectively according to pelvic vs no pelvic uptake status.

A very recent retrospective study (ONCO-GF) reported a PET/CT sensitivity in identifying PA lymph nodes metastases of 23.5%. Among 151 patients with negative PA lymph node at PET/CT, 26 (17,2%) had histological proven metastases including 21 with macroscopic lesions and the percentage increased considering patients with positive pelvic uptake (18,1%) confirming our observation. The authors conclude that para-aortic surgical staging contributes significantly to individualise the radiation treatment plan especially in patients with positive pelvic uptake [35]. FRANCOGYN study including 647 patients with LACC treated by concurrent chemoradiation therapy (CRT) and no evidence of para-aortic metastasis on pre-operative imaging work-up, confirmed in multivariate model analysis surgical staging as an independent prognostic factor for Disease Free Survival [36]. On the other hand, a retrospective study from Mayo Clinic registry including 148 patients did not find differences comparing surgical versus radiological staging (mainly based on CT, MRI and PET combination) in 5-year PFS (HR 1.11, 95% CI 0.54–2.30, *p* = 0.77) and 5-year OS (HR 1.02, 95% CI 0.46–2.29, *p* = 0.96). The presence of PA lymph nodes metastasis significantly predicted unfavourable PFS (HR 2.76, 95% CI 1.23–6.18, *p* = 0.01) and OS (HR 3.46, 95% CI 1.40–8.55 [37]. More consistent data will be forthcoming in large trial of surgical vs radiological staging. A prospective study, including 600 patients with stage IB2- IVA cervical cancer, is ongoing in order to evaluate the impact on survival of standard chemoradiotherapy treatment based only on ^18^F-FDG PET/CT staging compared to an adapted treatment based on PA lymph node dissection staging [38]. However, considering our results and previous published data, surgical lymph node dissection could be discussed in patients without involved pelvic lymph nodes on ^18^F-FDG PET/CT, considering the very low false negative rate in this case. On the other hand, in case of ^18^F-FDG PET/CT uptake in the pelvic region, in particular if bilateral, the risk of FN findings in PA lymph node is higher and surgical dissection could be preferred.

Our study presents several limitations, in particular the retrospective nature, the heterogeneity of the PET/CT scanners employed and the lack of a standardized protocol for images acquisition. Furthermore, for strength conclusions on the impact of TOF technique a direct comparison of images derived from the same patient with and without TOF reconstruction method should be performed through a proper trial. On the other hand, the impact of PET/CT device has been tested in a real clinical setting, in a wide sample size population with histological confirmation, and in comparable patient populations. At this moment the increasing availability of Digital PET scanner will probably change the scenario and a direct comparison among different reconstruction methods will be the next step to evaluate the impact of technological development in this clinical setting. Finally, patients with positive PA at ^18^F-FDG PET/CT have not been included in the study and the analysis of true or false positive findings in the PA region has not been performed because a reliable confirmation by histology would not have been available being the patient directly sent to extended EBRT protocol.

## Conclusions

The FN rate in PA lymph nodes detection in LACC is high, independently from PET/CT technology. Surgical staging should be preferred in case of negative pre-surgical imaging in the PA region, in particular in case of ^18^F-FDG PET/CT abnormal uptakes in pelvic lymph nodes. However, the FN rate in PA lymph nodes detection decreases dramatically in case of negative PET/CT in pelvic region and surgical staging could be discussed in this case. Prospective trials are mandatory to validate this approach.

## Data Availability

The datasets generated and/or analysed during the current study are not publicly available due privacy rules but are available from the first author on reasonable request at the following email address Sebastien.GOUY@gustaveroussy.fr.

## Abbreviations

^18^F-FDG: ^18^F- Fluorodeoxyglucose (FDG)
PET/CT: Positron Emission Tomography /Computed Tomography
CT: Computed Tomography
MRI: Magnetic Resonance Imaging
FIGO: International Federation of Gynecology and Obstetrics
LACC: Locally advanced cervical cancer
PA: Para-aortic
FN: False Negative
TOF: Time of Flight
EBRT: External Beam Radiation Therapy
GR: Gustave Roussy
BMI: Body Mass Index
CONSORT: Consolidated standards of reporting Trials
SUV: Standardized Uptake Value
